# Identification of Novel Inhibitors Targeting SGK1 via Ensemble-Based Virtual Screening Method, Biological Evaluation and Molecular Dynamics Simulation

**DOI:** 10.3390/ijms23158635

**Published:** 2022-08-03

**Authors:** Hui Zhang, Chen Shen, Hong-Rui Zhang, Hua-Zhao Qi, Mei-Ling Hu, Qing-Qing Luo

**Affiliations:** College of Life Science, Northwest Normal University, Lanzhou 730070, China; 2019212344@nwnu.edu.cn (C.S.); 2020212626@nwnu.edu.cn (H.-R.Z.); 2021212773@nwnu.edu.cn (H.-Z.Q.); 15693772469@163.com (M.-L.H.); luoqingqing1024@163.com (Q.-Q.L.)

**Keywords:** SGK1 inhibitor, virtual screening, biological evaluation, molecular dynamics

## Abstract

Serum and glucocorticoid-regulated kinase 1 (SGK1), as a serine threonine protein kinase of the AGC family, regulates different enzymes, transcription factors, ion channels, transporters, and cell proliferation and apoptosis. Inhibition of SGK1 is considered as a valuable approach for the treatment of various metabolic diseases. In this investigation, virtual screening methods, including pharmacophore models, Bayesian classifiers, and molecular docking, were combined to discover novel inhibitors of SGK1 from the database with 29,158 compounds. Then, the screened compounds were subjected to ADME/T, PAINS and drug-likeness analysis. Finally, 28 compounds with potential inhibition activity against SGK1 were selected for biological evaluation. The kinase inhibition activity test revealed that among these 28 hits, hit15 exhibited the highest inhibition activity against SGK1, which gave 44.79% inhibition rate at the concentration of 10 µM. In order to further investigate the interaction mechanism of hit15 and SGK1 at simulated physiological conditions, a molecular dynamics simulation was performed. The molecular dynamics simulation demonstrated that hit15 could bind to the active site of SGK1 and form stable interactions with key residues, such as Tyr178, ILE179, and VAL112. The binding free energy of the SGK1-hit15 was −48.90 kJ mol^−1^. Therefore, the identified hit15 with novel scaffold may be a promising lead compound for development of new SGK1 inhibitors for various diseases treatment.

## 1. Introduction

The phosphatidylinositol 3-kinase (PI3K) signaling pathway mediates a range of fundamental cellular processes [1]. Serum and glucocorticoid-regulated kinase 1 (SGK1), as a serine/threonine kinase, is as an effector of the PI3K signaling pathway, which regulates various cellular enzymes, transcription factors, ion channels, transporters, and cell proliferation and apoptosis [2,3,4]. SGK1 is activated by phosphorylation at specific residues in the protein structure, most notably Ser422 and Thr256 [5]. Overexpression of SGK1 has been found to be associated with a variety of pathophysiological processes, such as obesity, diabetes, ischemia, inflammation, cardiac arrhythmia disorders, neurodegenerative disorders, and various tumors [6,7]. SGK1 works under the control of acute transcription by several stimuli, including serum and glucocorticoids [8,9]. Given the importance of SGK1 in various diseases, the development of potent and selective small-molecule inhibitors targeting SGK1 is an urgent task in the field of drug discovery.

Presently, extensive efforts have been focused on the development and research of SGK1 inhibitors. Hundreds of SGK1 inhibitors with various scaffolds have been reported, such as GSK650394, EMD638683, TOFACITINIB, SGK1-IN-2, and SGK1-IN-1 [10,11,12,13,14]. Unfortunately, among these reported SGK1 inhibitors, none of them have successfully entered the market. In addition, although some inhibitors have entered into clinical trials, some side effects appeared in clinical application, such as metabolic toxicity, hepatotoxicity and carcinogenicity reported in DAILYMED (https://dailymed.nlm.nih.gov/dailymed/index.cfm, accessed on 6 May 2021). Therefore, there is a high demand for development of novel potent and selective SGK1 inhibitors with high potency and specificity to overcome these restrictions, which could provide more lead candidates for various SGK1-related disease treatment.

**Figure 1:**
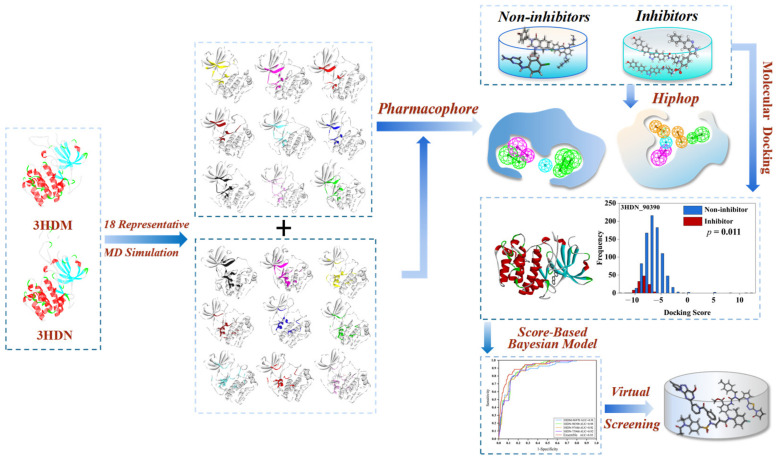
The ensemble-based virtual screening strategy was developed for discovering potent inhibitors targeting for SGK1.

In order to identify novel compounds capable of inhibiting SGK1 activity, a computer-aided high-throughput virtual screening technique was carried out. Computer-aided drug design (CADD) virtual screening has been widely used for the discovery of lead compounds with new scaffolds of specified targets from large chemical databases [15,16]. Among these CADD methods, the pharmacophore-based, molecular docking, and machine learning methods have been prevalently applied in the discovery of novel chemicals binding to an especial target [17,18,19,20,21,22]. Presently, more than 100 SGK1 inhibitors have been reported, which have provided a basis for the construction of ligand-based pharmacophore and machine learning prediction models. In addition, two cocrystallized structures of SGK1 protein complexed with different ligands (PDB: 3HDM and 3HDN) have been reported, which have provided a foundation for molecular docking and structure-based pharmacophore modeling. Therefore, in this investigation, the ligand-based pharmacophore, structure-based pharmacophore, Bayesian classifier, and molecular docking methods were used to establish the virtual screening platform for the SGK1inhibitors. Then, the retrieved molecules were further filtered by the rule of five (ROF), Veber’s rule, PAINS, ADME/T properties, and various toxicities [23]. The potential active compounds were selected and purchased for an enzyme activity test. Finally, the binding mode, stability, and molecular interaction pattern of the hit molecules and SGK1 at simulated physiological conditions were further probed by molecular dynamic (MD) simulations [24]. The specific technology roadmap of virtual screening of SGK1 inhibitors in this study is shown in Figure 1.

## 2. Results and Discussion

### 2.1. Representative Protein Conformer Selection Using Molecular Dynamics Simulation

**Figure 2:**
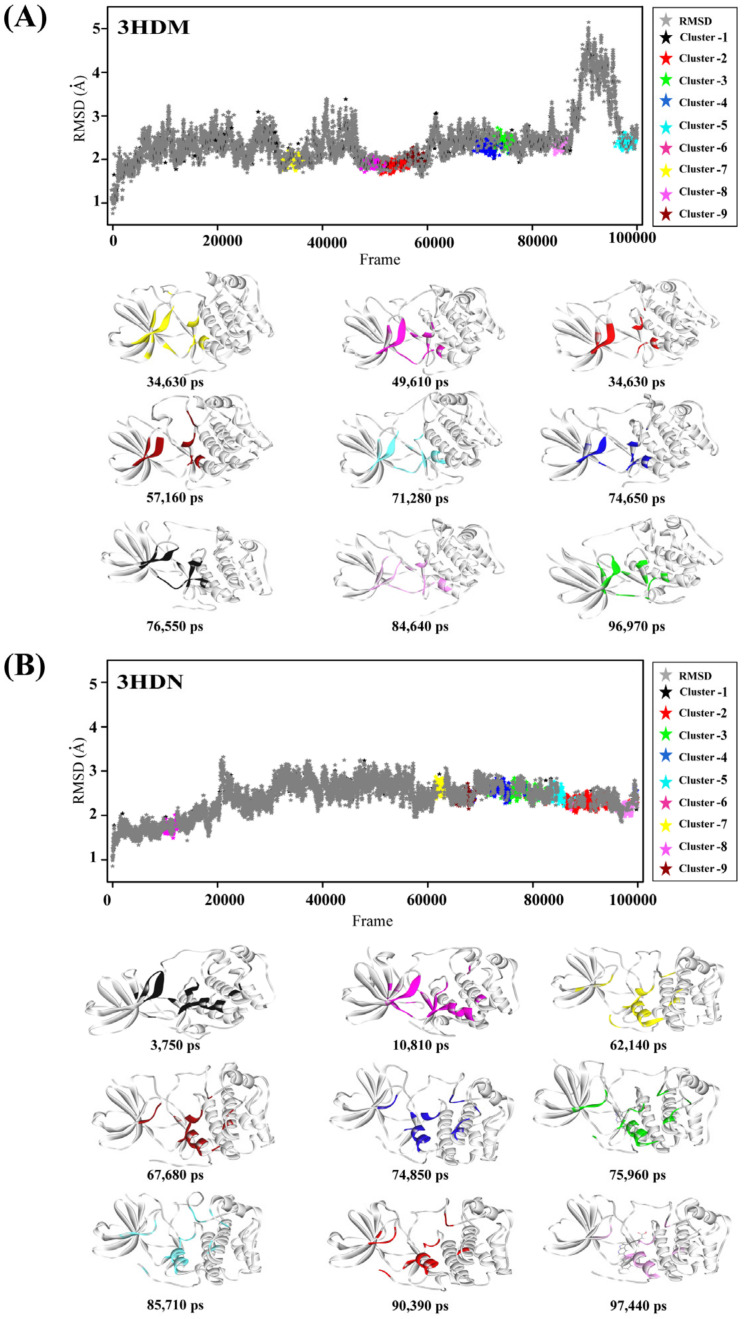
Representative protein conformers based on 3HDM (**A**) and 3HDN (**B**) were extracted from the MD trajectory by using structural clustering.

The binding mode of a crystallographic structure is static, whereas the structure of a receptor–ligand complex is flexible in the solvent. The crystal structures as well as protein conformers generated by the molecular dynamics simulation can increase the enrichment of known inhibitors. Therefore, in this investigation, the computational structures extracted from MD simulations were applied to probe protein-structure flexibility. 3HDM and 3HDN were selected as the initial structures for MD simulations, and various conformations of protein structure were generated during the 100 ns MD simulations. Then, these conformations were clustered according to their RMSD. Nine clusters for each initial structure were obtained. One representative structure was extracted from each of cluster. Finally, nine representative protein conformers based on 3HDM were obtained, including 3HDM_53690, 3HDM_74650, 3HDM_71280, 3HDM_96970, 3HDM_49610, 3HDM_34630, 3HDM_84640, 3HDM_57160, and 3HDM_76550. Nine representative protein conformers based on 3HDN were selected, such as 3HDN_90390, 3HDN_75960, 3HDN_74850, 3HDN_85710, 3HDN_10810, 3HDN_62140, 3HDN_97440, 3HDN_67680, and 3HDN_3750. Finally, 18 representative protein conformers were represented, as shown in Figure 2.

### 2.2. Common Feature Pharmacophore Modeling and Validation

**Figure 3:**
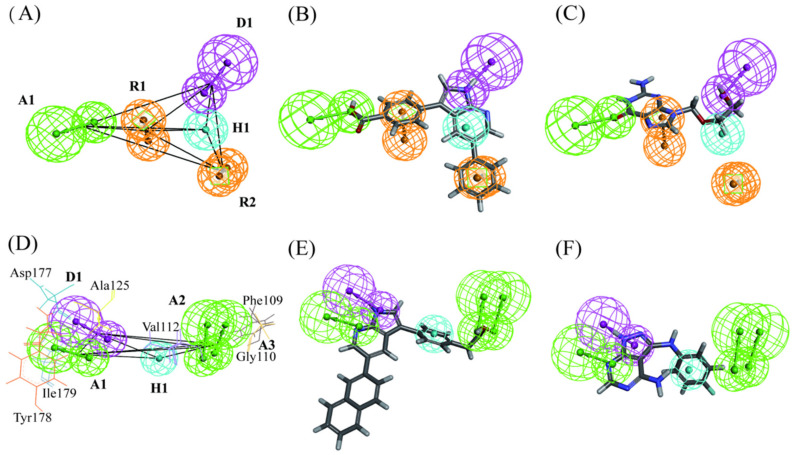
The best pharmacophore models of SGK1 inhibitors. (**A**) The 3D spatial relationship and geometric parameters of Hypo1. (**B**) The Hypo1 aligned with cpd1 (IC_50_ = 0.04 μM). (**C**) The Hypo1 aligned with lower activity of cpd6 (IC_50_ = 6.78 μM). (**D**) The 3D spatial relationship and geometric parameters of Hypo2. (**E**) The Hypo2 aligned with GMG. (**F**) The Hypo2 mapped onto the compound with lower activity (IC_50_ = 2.70 μM).

Six SGK1 inhibitors with different activity values were selected for the common feature pharmacophore model generation. Finally, a total of ten pharmacophore models with various features were obtained. The Hypo1, containing one hydrogen bond acceptor, one hydrogen bond donor, one hydrophobic feature, and two ring-aromatic rings, was considered as the best pharmacophore model (Figure 3A). The 3D space and distance constraints of these pharmacophore features are displayed in Figure 3A. The most potent inhibitor (IC_50_ = 0.04 μM) was mapped very well with these features of the Hypo1 (fit value = 4.99) (Figure 3B). The compound with lower activity (IC_50_ = 6.78 μM) cannot match with some features of Hypo1 (fit value = 1.23) (Figure 3C). The above results indicated that Hypo1 could reflect essential chemical characteristics for an effective SGK1 inhibitor.

Subsequently, the 119 SGK1 known inhibitors were mapped onto the features of Hypo1. The results exhibited that among these 119 inhibitors, the fit values of 97 agents were greater than 3.5. In addition, the Güner-Henry (GH) score method was further carried out to verify the reliability of the Hypo1 model. The decoy set, comprising of 119 active agents (SGK1 inhibitors) and 881 inactive agents (noninhibitors), was used to evaluate the discriminative ability of the Hypo 1. As can be seen from Table 1, among 1000 compounds, 135 agents, comprising of 97 active agents and 38 inactive agents, were predicted as actives. The yield of actives was 71.85%, the ratio of actives was 81.51%, the enrichment factor was 6.04, and the goodness of hit score was 0.71. This suggested that the Hypo1 had preferable performance of distinguishing active compounds from inactive compounds.

### 2.3. Structure-Based Pharmacophore Generation and Validation

Structure-based pharmacophore modeling is based on the actual interaction between receptor and ligand to produce pharmacophore models. In this investigation, the two crystal structures of SGK1 (PDB code: 3HDM and 3HDN) extracted from PDB and 18 representative protein conformers extracted from MD simulations were used to produce structure-based pharmacophore models. Finally, 200 pharmacophore models were obtained. It was reported that the residues of Gly107, Val112, Lys127, Asp177, Tyr178, and Ile179 played important roles in identification of SGK1inhibitors. From further careful analysis of these 200 pharmacophore models, it was found that the features of Hypo2 based on 3HDN, comprising of three hydrogen bond acceptors, one hydrogen bond donor, and one hydrophobic feature, could interact with these key residues. As can be seen from Figure 3D, the hydrogen bond acceptor features of A1, A2, and A3 formed hydrogen-bonding interactions with Tyr178, Ile179, Gly117, and Phe109, respectively. The hydrophobic feature (H1) formed a hydrophobic interaction with the Val112. The hydrogen bond donor feature (D1) was complementary to the residue Asp177. As exhibited in Figure 3E, the native ligand GMG binding to 3HDN was mapped well with these features of the Hypo2 (fit value  =  4.57). The compound with lower activity (IC_50_  =  2.70 μM) cannot match with most of the features of Hypo 2 (fit value  =  2.13) (Figure 3F).

Moreover, the 119 inhibitors were matched with these features of Hypo2. The results represented that the fit values of the 92 inhibitors were greater than 3.5, which indicated that the Hypo2 contained the necessary features of SGK1 inhibitor. Furthermore, the discriminative performance of the Hypo2 was validated by the GH score method. As can be seen from Table 1, among 1000 compounds, 118 agents, containing 92 active agents and 26 inactive compounds, were predicted as hits. The yield of actives was 77.97%, the ratio of actives was 77.31%, the enrichment factor was 6.55, and the goodness of hit score of Hypo2 was 0.76. This suggested that the Hypo2 had a good ability of distinguishing inhibitors from noninhibitors.

### 2.4. Molecular Docking Method Selection

The two crystal structures of SGK1 (PDB code: 3HDM and 3HDN) extracted from PDB were used as the receptor structures to perform molecular docking study. The native ligands extracted from 3HDM and 3HDN were redocked into the active sites by the CDOCKER, Gold, AutoDock, and AutoDock Vina. The RMSD between the docked and original poses is displayed in Table 2. Among these four molecular docking methods, the AutoDock software gave the minimum average RMSD of 0.62 Å, suggesting that the method could generate a correct pose. Therefore, the AutoDock method would be employed for molecular docking to improve the efficiency of virtual screening.

### 2.5. Performance of Bayesian Models Based on Molecular Docking Score

**Figure 4:**
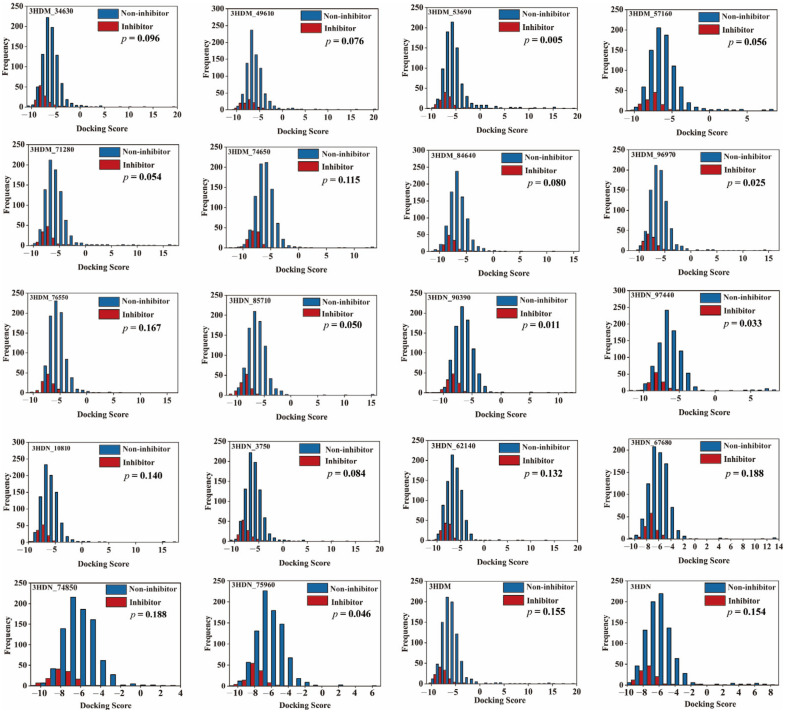
Distributions of the docking scores of the inhibitors/noninhibitors for each SGK1 protein.

The 1000 compounds, including 119 inhibitors and 881 noninhibitors, were used as the data set. The 20 protein conformers, including two crystal structures of SGK1 (PDB code: 3HDM and 3HDN) extracted from PDB and eighteen representative structures extracted from molecular dynamics (MD) simulations, were used in this study. Then, all compounds were docked into the 20 protein conformers by using the AutoDock. The docking scores for each protein–ligand complex were given in Table S1. Then, the Student’s *t*-test was carried out to evaluate the significant differences between inhibitors and non-inhibitors. The *p*-value was used in statistical hypothesis testing to quantify the statistical significance of sample. The *p*-value less than 0.05 indicated that the data were statistically significant. The distributions of these 20 docking scores for inhibitors and non-inhibitors are displayed in Figure 4. As can be seen from Figure 4, among these 20 complexes, the *p*-values of the docking scores based on 3HDN_90390, 3HDM_96970, 3HDN_97440, and 3HDN_75960 were less than 0.05, indicating that the distributions of the four docking scores in the means of inhibitors and non-inhibitors were significantly different. Thus, the docking scores based on the 3HDN_90390, 3HDM_96970, 3HDN_97440, and 3HDN_75960 were used to establish the Bayesian classification model for the SGK1 inhibitor.

**Figure 5:**
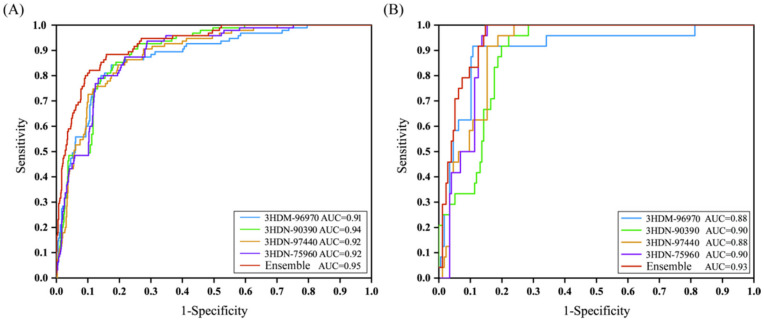
The ROC curve of the naive Bayesian classifier. (**A**) Training set; (**B**) test set.

The 1000 compounds with the docking scores were randomly split into a training set with 800 compounds (80% of the data: 95 inhibitors and 705 noninhibitors) and a test set with 200 agents (20% of the data: 24 inhibitors and 176 noninhibitors). The Bayesian classification models were established with using the training set and docking scores. Firstly, the Bayesian classification models were constructed based on each of four docking scores. The detailed statistical results of the 5-fold cross-validation for the training sets are listed in the Table 3. As can be seen from Table 3, for the four complexes, the Bayesian classification model gave SE ranging from 0.84 to 0.87, SP between 0.80 and 0.82, *Q* ranging from 0.81 to 0.82, MCC values between 0.46 and 0.48, and Roc score values between 0.88 and 0.90. In addition, the prediction power of these Bayesian classification models was further measured by AUC values. The AUC values of the training set based on four protein structures are displayed in Figure 5A. As can be seen from Figure 5A, among these established Bayesian classification models based on 3HDN-90390, 3HDM-96970, 3HDN-97440, and 3HDN-75960, the 3HDN-90390 gave a better performance with the AUC value of 0.94. Then, another Bayesian classification model was established by combination of the four docking scores (Model-ensemble). As shown in Table 3, the ensemble model generated SE of 0.88, SP of 0.83, *Q* of 0.83, MCC of 0.52, and Roc score of 0.93. As exhibited in Figure 5A, the AUC value for the ensemble model was 0.95. Obviously, among these established prediction models, the ensemble model represented the best performance for discriminating the inhibitors from noninhibitors.

In addition, an external test set, comprising of 24 inhibitors and 176 noninhibitors, were employed to assess the predictive power of the established classification models. The detailed prediction results of the test set were summarized in Table 3. As can be seen from Table 3, among these established prediction models based on the docking scores, the ensemble model represented the best prediction performance, which gave SE of 1.00, SP of 0.84, *Q* of 0.86, MCC of 0.62, and Roc score of 0.95. Furthermore, the ensemble model produced an AUC value of 0.93 for the external test set (Figure 5B). In summary, the above results suggested that the ensemble model based on the combination of the four docking scores represented the best performance for SGK1 inhibitors’ identification, which would be used for virtual screening to identify potential SGK1 inhibitors from large database.

### 2.6. Virtual Screening

The virtual screening models of SGK1 inhibitors, containing the common feature pharmacophore model (Hypo1), structure-based pharmacophore model (Hypo2), and Bayesian prediction model (Model-ensemble), have been successfully established. The Autodock method was selected for molecular docking study. The virtual screening of SGK1 inhibitors was performed based on the workflow exhibited in Figure 1. The detailed virtual screening process was following as: (1) the common feature pharmacophore (Hypo1) was used to retrieve the BioDiversity database with 29,158 compounds, and 1678 compounds with fit values greater than 3.5 were remained. (2) The structure-based pharmacophore (Hypo2) was further used to filter these compounds retrieved by Hypo1, and 161 compounds with fit values greater than 3.5 were remained. (3) The remaining 161 molecules were assessed by the Bayesian model (Model-ensemble) based on the docking scores of four representative structures. A total of 100 agents were identified as SGK1 inhibitors. (4) Depth visual inspection of the 100 molecules. Six residues, comprising of Gly107, Val112, Lys127, Asp177, Tyr178, and Ile179, were considered as crucial points for visual inspection. (5) The top-ranking 60 compounds were subjected to PAINS, drug-likeness, and ADME/T properties analysis. (6) 28 compounds exhibited better properties, as summarized in Table S2, and were further purchased for enzyme activity test. 

### 2.7. Kinase Inhibition Results

The effects of 28 hits on SGK1 enzyme were detected by ADP-GLO method. Staurosporine (STSP) was selected as the positive control. The 28 compounds were tested at the initial concentration of 10 μM. The results of the SGK1 inhibitory activities of these hits are listed in Table S3. Among these 28 hits, hit15 exhibited the best inhibitory effect on SGK1, which gave inhibition rate of 44.79% at the concentration of 10 µM. For the positive control group, the STSP represented an inhibition rate of 99.16% against SGK1 at the concentration of 10 µM. Obviously, hit15 displayed lower inhibitory activity on SGK1 than that of the STSP. In order to investigate the interaction mechanism differences between SGK1-hit15 and SGK1-STSP, the molecular dynamics simulation was performed. 

### 2.8. Molecular Dynamics Simulation and Analysis

The results of the kinase inhibition indicated that the hit15 with novel scaffold is a potential inhibitor of SGK1. The molecular dynamics simulations and MM-PBSA calculations were performed to probe the stability and molecular interaction pattern of hit15 and SGK1. The STSP, and GMG binding to the 3HDN crystal structure were used as the reference molecules. The docking conformations of STSP, GMG, and hit15 generated by the Autodock were selected for 100 ns MD simulations. 

#### 2.8.1. Structural Deviations and Compactness Analysis

**Figure 6:**
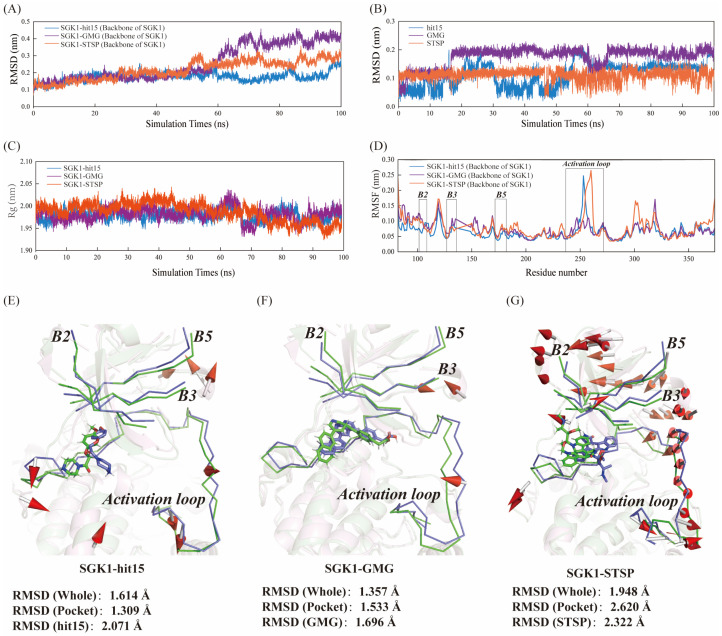
Dynamics of hit15, GMG, and STSP binding to the SGK1 active site. (**A**) Comparison of orientations of protein backbone for three SGK1-ligand systems. (**B**) Comparison of orientations of ligands for the three SGK1-ligand systems. (**C**) Time evolution of radius of gyration (Rg) values during 100 ns MD simulation. (**D**) Cα atomic fluctuations root-mean-square fluctuation (RMSF) plots. Blue, purple, and red represent the SGK1-hit15, SGK1-GMG, and SGK1-STSP, respectively. Root-mean-square deviation (RMSD) plots as a function of time. (**E**–**G**) represented the structural superposition before (blue) and after (green) MD for SGK1-hit15 (**E**), SGK1-GMG (**F**), and SGK1-STSP (**G**). A porcupine plot was generated and the movements of flexible parts of the SGK1 indicated with red arrows, and cone represents the direction.

The dynamic stability of the complex was elucidated by calculating the RMSD values of the protein backbone (Figure 6A) and ligands (Figure 6B). For the protein backbone, as represented in Figure 6A, the RMSD values became stable after 65 ns for structure. For the ligands, as displayed in Figure 6B, the RMSD values became stable after 70 ns. The structural integrity and compactness of the protein-ligand in a biological system was evaluated by the parameter of Rg. As shown in Figure 6C, the average Rg values for hit15, GMG, and STSP were 1.97, 1.98, and 1.95 nm, respectively. For the three systems, Rg values fluctuated around 1.90–2.05 nm. It indicated that the three systems studied in this investigation were relatively compacted. It was reported that the active site of SGK1 involved the B2, B3, and B5 regions of secondary structure and the activation loop. As exhibited in Figure 6D, the residues for the three systems showed similar fluctuations, which indicated that that hit15 could form an interaction with amino acids in the active pocket of SGK1. Especially, the activation-loop region displayed greater fluctuations. Furthermore, the structural superposition of SGK1-ligand before (blue) and after (green) MD was represented in Figure 6E–G. As given in Figure 6E–G, the B2, B3, and B5 regions and the activation loop region displayed greater displacement changes. The movements of flexible parts of the SGK1 are indicated with red arrows. For SGK1-hit15 (Figure 6E), the RMSD values of whole SGK1 and active pocket were 2.614 Å and 1.309 Å, respectively. For SGK1-GMG (Figure 6F), the RMSD values of whole SGK1 and active pocket were 1.357 Å and 1.533 Å, respectively. For SGK1-STSP (Figure 6G), the RMSD values of whole SGK1 and active pocket were 1.948 Å and 2.620 Å, respectively. From comparative analysis of the superimposed between before and after simulation, it was observed that the structures of SGK1 and the active pocket produce displacement changes. 

**Figure 7:**
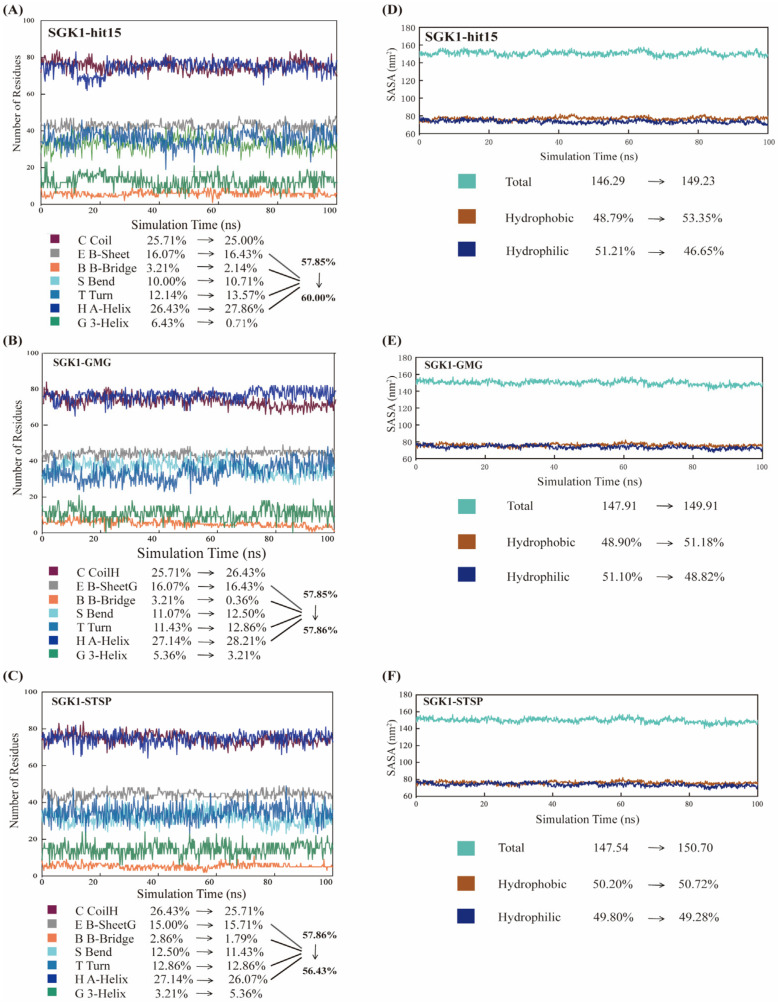
Molecular dynamics results of SS and SASA during 100 ns MD simulation. (**A**–**C**) represented the SS changes during MD simulation for SGK1-hit15 (**A**), SGK1-GMG (**B**), and SGK1-STSP (**C**). (**D**–**F**) exhibited the total, hydrophobic, and hydrophilic area changes during MD simulation for SGK1-hit15 (**D**), SGK1-GMG (**E**), and SGK1-STSP (**F**).

In addition, the SS and SASA were further used to evaluate the effects of ligands on the stability of protein structure. As represented in Figure 7A–C, the α-Helix (H), β-Sheet (E), β-Bridge (B), and Turn (T) SS were favorable for protein structural stability. The percentage of H + E + B + T was changed from 57.85% to 60.00% for hit15 (Figure 7A), from 57.85% to 57.86% for GMG (Figure 7B), and 57.86% to 56.43% for STSP (Figure 7C). Moreover, for the three systems, as displayed in Figure 7D–F, the hydrophobic area was increased, while the hydrophilic area was decreased. The above results indicated that the hit15, similar to GMG and STSP, could form a stable system with SGK1. 

#### 2.8.2. Hydrogen Bond Analysis

**Figure 8:**
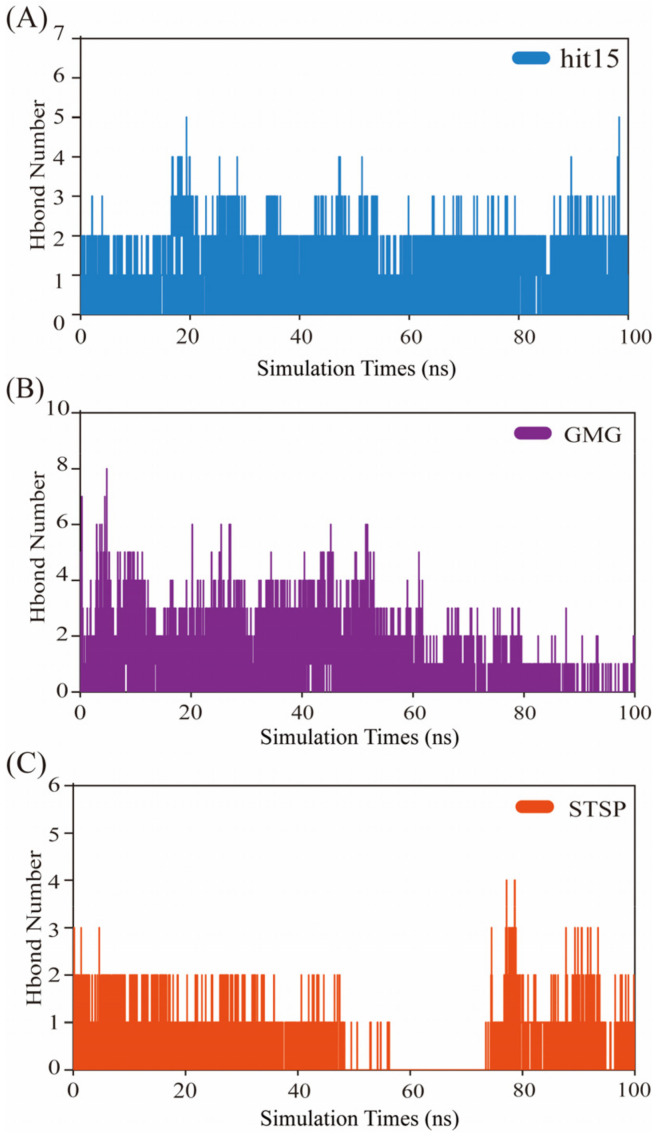
Number of hydrogen bond interactions formed during MD simulation in the case of SGK1-hit15 (**A**), SGK1-GMG (**B**), and SGK1-STSP (**C**).

Hydrogen bond stability was applied for validation of system stability. The H-bond numbers of SGK1-hit15, SGK1-GMG, and SGK1-STSP were calculated. The geometric criteria for hydrogen bond formation between acceptor and donor were kept less than 3.5 Å, and the angle between acceptor and donor were set as 30 Å. The number of hydrogen bonds formed in SGK1-ligand complexes during the 100 ns trajectory are depicted in Figure 8. For the SGK1-hit15 system (Figure 8A), a maximum of five hydrogen bonds were observed, which proved that it was an inhibitor of SGK1. For the SGK1-GMG system (Figure 8B), a maximum of three hydrogen bonds were formed. The hydrogen bonds’ formation in SGK1-GMG was unstable, and fewer hydrogen bonds were observed during the last 20 ns stimulations. For the SGK1-STSP system (Figure 8C), a maximum of three hydrogen bonds were observed. The hydrogen bonds’ formation was unstable, which did not appear during the 58–77 ns stimulations. Therefore, among these three systems, the SGK1-hit15 system could form stable hydrogen bond interactions during the stimulations.

#### 2.8.3. The Interaction Mechanism between SGK1 and hit15 Simulated by Molecular Dynamics

**Figure 9:**
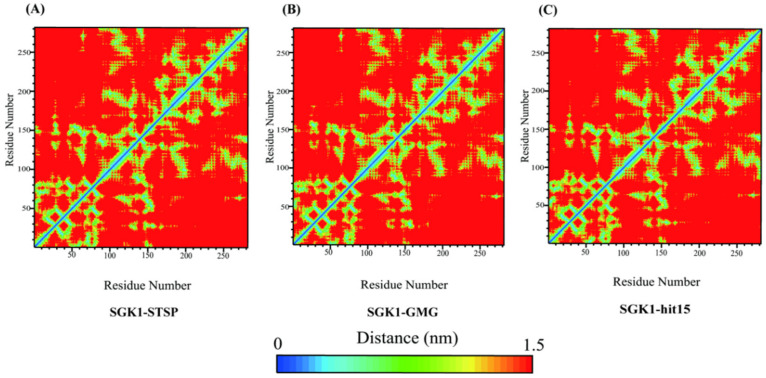
Molecular dynamics results of tertiary contact map for SGK1-STSP (**A**), SGK1-GMG (**B**), and SGK1-hit15 (**C**).

**Figure 10:**
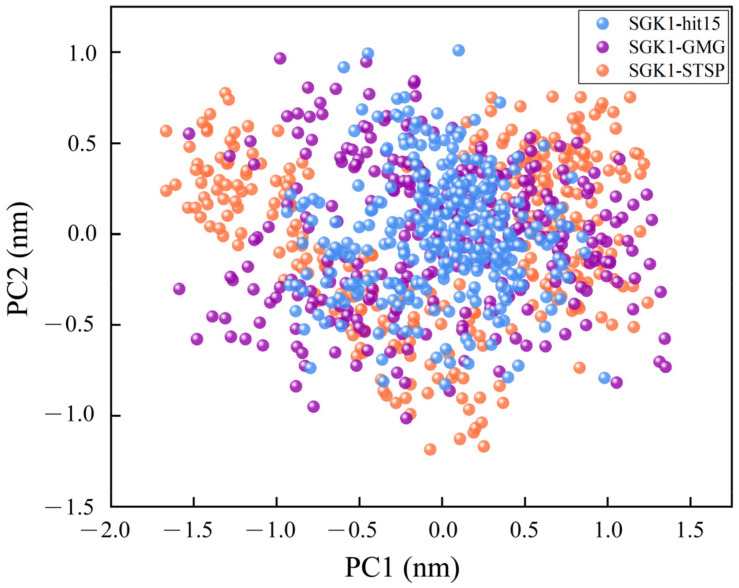
Molecular dynamics results of PCA analysis from the last 5 ns trajectories. Projection of the motion of SGK1-hit15, SGK1-GMG, and SGK1-STSP in phase space along the PC1 and PC2.

In order to investigate the difference of motion between hit15 and reference compounds (GMG and STSP), the contact maps were produced by using conformations extracted from the equilibrium trajectories. The contact maps depicted the extent of contact among residues by using color-coded modes. As exhibited in Figure 9, it was found that the contact map of hit15 was very similar to that of GMG and STSP, suggesting that hit15 could bind to the SGK1 active site. Principal component analysis (PCA) analysis is used to reveal the dominant motion modes in an MD trajectory. The dominant motion modes’ similarity between two MD trajectories was obtained by comparing PC projections. As shown in Figure 10, the three systems had similar dominant motion modes with −2.0 < PC1 < 1.5 nm and −1.5 < PC2 < 1.0 nm.

**Figure 11:**
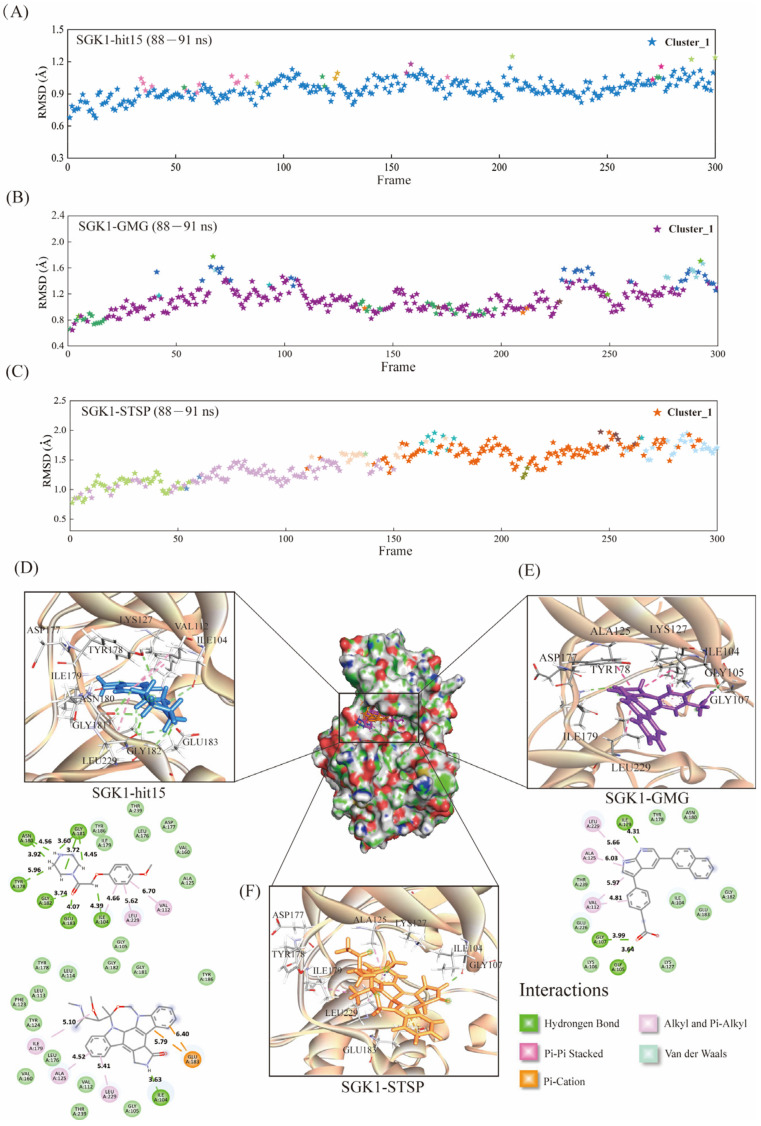
Molecular dynamics results of clustering and binding mode analysis. (**A**) Conformation clustering from the last 3 ns of SGK1-hit15. The Cluster_1 was colored by blue. (**B**) Conformation clustering from the last 3 ns of SGK1-GMG. The Cluster_1 was colored by purple. (**C**) Conformation clustering from the last 3 ns of SGK1-STSP. The Cluster_1 was colored by red. (**D**–**F**) represents the binding modes of SGK1-hit15 (**D**), SGK1-GMG (**E**), and SGK1-STSP (**F**). Hydrogen bonds are depicted as green dashed lines, Alkyl and Pi-Alkyl are represented as pink dashed lines, Pi-Pi Stacked are depicted as purple-pink dashed lines, Pi-Cation are exhibited as orange dashed lines.

In order to obtain the stable binding modes, the Bitclust trajectory method was used to select the representative conformation for the three systems. The structural frames of the three systems were clustered according to RMSD values. As shown in Figure 11A–C, the frames in the largest cluster_1 were determined for the SGK1-hit15 (Figure 11A), SGK1-GMG (Figure 11B), and SGK1-3TSP (Figure 11C) during 88–91 ns trajectory. The representative structures of the SGK1-hit15 (88.74 ns), SGK1-GMG (90 ns), and SGK1-3TSP (90.22 ns) were extracted from cluster_1 for molecular interaction mechanism analysis (Figure 11D–F). For the SGK1-hit15 system, as exhibited in Figure 11D, residues of Gly181_3.60/3.72/4.45 Å_, Asn180_3.92/4.56 Å_, Tyr178_5.96 Å_, Gly182_3.74 Å_, Glu183_4.07 Å_, and Ile104_4.39 Å_ formed hydrogen bonds with hit15; residues of Ile104_4.66 Å_, Leu229_5.62 Å_, and Val112_6.70 Å_ were observed to be participating in hydrophobic interactions; and residues of Tyr186, Ile179, Thr239, Leu176, Asp177, Val160 and Ala125 were found to be participating in van der Waals interactions. For the SGK1-GMG system, as represented in Figure 11E, residues of Leu229_5.66 Å_, Ala125_6.03 Å_, and Val112_5.97/4.81 Å_ formed hydrophobic interactions with GMG; residues of Ile179_4.31 Å_, Gly107_3.99 Å_, and Gly105_3.64 Å_ formed hydrogen bonds with GMG; and residues of Tyr178, Asn180, Thr239, Ile104, Glu183, Gly182, Glu226, Lys106, and Lys127 interacted with GMG as van der Waal interactions. For the STSP, as displayed in Figure 11F, the hydrogen bond was formed with Ile104_3.64 Å_, hydrophobic interactions were observed between STSP and residues of Ile179_5.10 Å_, Ala125_4.52 Å_, and Leu229_5.41 Å_; electrostatic interactions were formed with Glu183_5.79/6.40 Å_; and Van der Waal interactions were observed between STSP and residues of Tyr124, Phe123, Ieu123, Tyr178, Ieu114, Gly182, Gly105, Gly181, Tyr186, Gly105, Thr239, Val112, Val160, and Leu176. Compared with the SGK1-STSP system, it was obviously found that electrostatic interaction did not appear in the SGK1-hit15 system. Moreover, the number of residues participating in hydrophobic and van der Waal interactions were relatively fewer in the SGK1-hit15 system. These may be the reasons that the kinase inhibition activity of STSP was stronger than that of hit15, which could provide theoretical guidance for hit15 modification in the future.

#### 2.8.4. Binding Free-Energy Calculation and Decomposition

**Figure 12:**
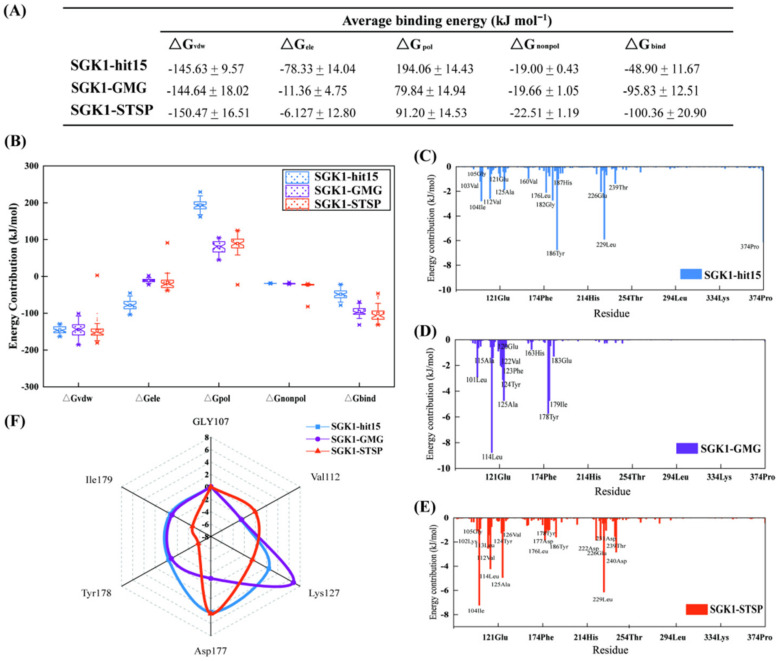
Binding free energy calculated and decomposed by MM/PBSA method. (**A**) The binding free energies of SGK1-hit15, SGK1-GMG, and SGK1-STSP. (**B**) The component energies of binding free energy for SGK1-hit15, SGK1-GMG, and SGK1-STSP. (**C**–**E**) indicate per-residue binding free-energy decomposition for SGK1-hit15 (**C**), SGK1-GMG (**D**), and SGK1-STSP (**E**). (**F**) The radar map of 6 important residues binding free-energy decomposition for SGK1-hit15, SGK1-GMG, and SGK1-STSP.

In this investigation, the binding free energy was effectively estimated by using the MM-PBSA method. For each system, 50 snapshots were extracted from structural ensemble recorded in the MD trajectory during the last 5 ns at interval of 100 ps. The binding free energy ΔG_bind_ for three systems is displayed in Figure 12A. The ΔG_bind_ for SGK1-hit15, SGK1-GMG, and SGK1-STSP were −48.90, −95.83, and −100.36 kJ mol^−1^, respectively. Obviously, the binding free energy of the SGK1-STSP was stronger than that of the SGK1-hit15. The tendency of binding free energies predicted were in line with the results of the kinase inhibition experimental test, which suggested that the free energy analysis used in this study was reasonable. The ΔG_bind_ was comprised of ΔG_vdw_, ΔG_ele_, ΔG_nonpo_, and ΔG_pol_. As can be seen from Figure 12B, the energies of ΔG_vdw_, ΔG_ele_, and ΔG_nonpol_ were favorable to inhibitor binding, but ΔG_pol_ was unfavorable to inhibitor binding. Among these systems, the ΔG_pol_ value for SGK1-hit15 was the greatest, which was the main reason why the ΔG_bind_ of SGK1-hit15 was weaker than that of the SGK1-STSP.

In addition, in order to identify potential hot residues, residue contributions and energy decomposition analyses for the three systems were performed (Figure 12C–E). In this study, residues with energy values less than −1.0 kcal mol^−1^ were considered to be important. For the SGK1-hit15 system, as exhibited in Figure 12C, 15 residues were found to be important, such as Val103, Ile104, Gly105, Val112, Glu121, Ala125, Val160, Leu176, Gly182, Tyr186, His187, Glu226, Leu229, Thr239, and Pro374. For the SGK1-GMG system, as represented in Figure 12D, residues of Leu101, Leu114, Ala115, Glu120, Val122, Phe123, Tyr124, Ala125, His163, Tyr178, Ile179, and Glu183 showed stronger interactions. For the SGK1-STSP system, as displayed in Figure 12E, residues of Lys102, Ile104, Gly105, Val112, Leu113, Leu114, Tyr124, Ala125, Val126, Leu176, Asp177, Tyr178, Tyr186, Asp222, Glu226, Leu229, Asp231, Thr239, and Asp240 were observed to be important. From the above results, it is clearly observed that the number of critical residues that specifically contributed to SGK1-STSP binding free energy were relatively higher than that of the SGK1-hit15. In addition, some critical residues, such as Ile104, Val121, Leu114, Leu229, and Asp240 that contributed to SGK1-STSP binding free energy were significantly stronger than that of the SGK1-hit15.

## 3. Materials and Methods

### 3.1. Crystal Structures Preparation

To date, only 2 co-crystallized receptor–ligand complexes (PDB: 3HDN and 3HDM) were reported, and extracted from RCSB Protein Data Bank (PDB, https://www.rcsb.org/, accessed on 10 June 2021). All hydrogen atoms were added, all water molecules were deleted, and the protein structures were optimized by Discovery Studio 3.5 (DS 3.5) with using CHARMm force field. Then, the 3HDN and 3HDM crystal structures were used as the initial structure for 100 ns MD simulations, respectively. The whole trajectories of the 3HDN and 3HDM were clustered according to their root mean square deviation (RMSD) values. Finally, nine clusters with the largest number of structures were obtained from each of 3HDN and 3HDM crystal structures. One protein conformer was randomly extracted from each cluster as a representative conformation. Finally, 18 representative conformations were obtained for further analysis.

### 3.2. Dataset Collection

A total of 119 SGK1 inhibitors with cell activities were collected from the ChemBel database. Compared with these SGK1 inhibitors, noninhibitors of SGK1 were reported to be relatively fewer. Therefore, in order to ensure that the collected agents had no biological activity for SGK1, those marketed drugs from Comprehensive Medicinal Chemistry (CMC) library that had been widely used in the clinic and had no any information about their kinase activity for SGK1 were chosen. Finally, 881 drugs were selected and considered as noninhibitors. The 3D structure of all the molecules was converted using the “Prepare Ligands” protocol in Discovery Studio 3.5 (Accelrys Inc., San Diego, CA, USA) (Inc., 2010) (http://accelrys.com/products/discovery-studio/, accessed on 4 July 2022). 

### 3.3. Pharmacophore Modeling

#### 3.3.1. Common Feature Pharmacophore Modeling

**Figure 13:**
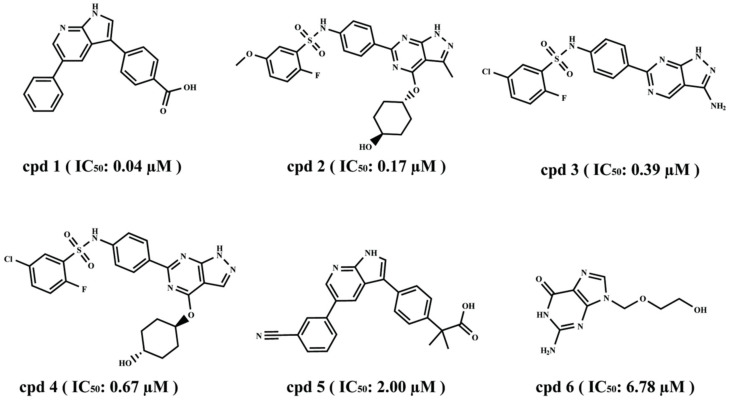
The chemical structures of SGK1 inhibitors together with their biological activity data (IC_50_) for HipHop running.

The common feature pharmacophore model was developed by using the HipHop algorithm implemented in the Accelrys DS 3.5 program package. Six well-known SGK1 inhibitors based on structural diversity were utilized for the Hiphop pharmacophore generation (Figure 13). These molecules were minimized using the CHARMm force filed. Among these compounds, the cpd 1 with the highest inhibition activity (IC_50_ = 0.04 μM) was chosen as the “reference compound”, the “Max-OmitFeat” and “Principal”, which were assigned as 0 and 2, respectively. Five features, including hydrogen bond acceptor, hydrogen bond donor, hydrophobic, hydrophobic-aliphatic, and ring-aromatic, were initially selected for the pharmacophore generation. The other parameters were set as default values. Finally, 10 common feature pharmacophore models were generated. 

#### 3.3.2. Receptor–Ligand Pharmacophore Modeling

The Receptor-Ligand Pharmacophore Generation tool of Discovery Studio v3.5 was employed for pharmacophore model generation. The 2 crystal structures (3HDN and 3HDM) extracted from RCSB Protein Data Bank and 18 representative structures extracted from MD trajectory were used to produce the receptor-ligand pharmacophore models. It was reported that residues of Gly107, Val112, Lys127, Asp177, Tyr178, and Ile179 played important roles in identification of SGK1inhibitors. According to the protein–ligand interactions, 10 receptor–ligand pharmacophore models for each crystal structure were generated. Finally, 200 pharmacophore models based on protein–ligand interactions were obtained. 

#### 3.3.3. Validation of Pharmacophore Models

The generated common feature pharmacophore models and receptor–ligand pharmacophore models were validated by Güner-Henry (GH) score method [25]. The 1000 agents, comprising of 119 SGK1inhibitors and 881 noninhibitors, were used as the decoy set. A GH score more than 0.70 indicated that the performance of pharmacophore model was perfect. The computational formula of GH method is given as bellow:

GH = [Ha/(4Ht × A)](3A + Ht)[1 − (Ht − Ha)/(D − A)](1)

The parameter of Ha represents the number of active hits; Ht means the total number of hits including active and inactive molecules; A depicts the number of active molecules in the decoy set; D expresses the total number of molecules in the decoy set.

### 3.4. Molecular Docking

In this study, the 2 crystal structures (3HDN and 3HDM) extracted from RCSB were applied for molecular docking. The native ligand binding to crystal structure was selected as the reference ligand. The active site was determined by a grid box sized 22 × 22 × 22 Å, which was large enough to overlay the native ligand-binding area at the active site. Four programs, including CDOCKER (DS3.5), Gold (version 5.1), AutoDock (version 4.2.6), and AutoDock Vina (version 1.1.2) [26,27,28], were employed for the molecular docking study. The 2 native ligands were extracted, and further redocked into the active sites. The RMSD value was used to measure the differences between the original crystallographic and docked poses. The program gave minimum average RMSD value was selected for final database screening. 

### 3.5. Bayesian Model

The naïve Bayes classifier, as a simple probabilistic machine learning method, has been widely used in drug development [29]. In this investigation, the docking scores calculated by the molecular docking software were used as the independent variable (X), and 1/−1 (1: SGK1 inhibitor, −1: noninhibitor) was used as the response variable (Y). The collected 1000 compounds were randomly split into a training set with 800 compounds (80% of the data: 95 inhibitors and 705 noninhibitors) and a test set with 200 agents (20% of the data: 24 inhibitors and 176 noninhibitors) by the “Generate Training and Test Data” protocol in Discovery Studio 3.5 (http://accelrys.com/products/discovery-studio/, accessed on 4 July 2022). The internal cross-validation was defined as 5. Then, the Bayesian classification model for SGK1 inhibitors were generated by using the Bayesian Model protocol in DS 3.5. 

The parameters based on TP (true positives), TN (true negatives), FP (false positives), and FN (false negatives) were applied to evaluate the performance of the established Bayesian model, such as sensitivity (SE (Equation (2))), specificity (SP (Equation (3))), overall prediction accuracy (*Q* (Equation (4))), and Mathews Correlation Coefficient (MCC (Equation (5))). In addition, the receiver operating characteristic curve (Roc) and area under Roc (AUC) were also used to evaluate the performance of Bayesian model.

(2) $$SE=\frac{TP}{TP+FN}\times 100\%$$

(3) $$SP=\frac{TN}{TN+FP}\times 100\%$$

(4) $$Q=\frac{TP+TN}{TP+TN+FP+FN}\times 100\%$$

(5) $$MCC=\frac{TP\times TN-FP\times FN}{\sqrt{\left(TP+FP)\times (TP+FN)\times (TN+FP)\times (TN+FN\right)}}\times 100\%$$

### 3.6. PAINS Filter, Drug-Likeness Assessment, and ADME/T Properties Prediction

In order to avoid false-positive compounds, the Pan Assay Interference Compounds (PAINS) screening was carried out by using online program “PAINS Remover” (http://www.cbligand.org/PAINS/) [30]. The drug-like properties were assessed by using the rule of five (ROF) and Veber’s rule [31,32]. The ADME/T properties were assessed by using the DS3.5 software. 

### 3.7. Kinase Activity Assay

The enzyme activity was measured by the ADP-Glo kinase assay [33], which was performed by the Topscience Biochemical Technology Company. The ADP-Glo assay is a chemiluminescence kinase assay, which detects the formation of ADP in kinase reaction. ADP converted to ATP was converted into light by Ultra-Glo™ Luciferase. The luminescence signal was positively correlated with kinase activity. The tested compounds were dissolved in DMSO, and then diluted to 10 µM. Firstly, 1 μL of tested compounds (10 μΜ), 2 μL of enzyme (2.5 ng/well), and 2 μL of substrate/ATP mix (50 μM ATP, 0.2 μg/μL ATK (PKB) Substrate) were added to 96-well plate and incubated at 25 °C for 60 min. Then, 5 μL of ADP-Glo™ Reagent was added to each well, and incubated at 25 °C for 60 min. 10 μL of Kinase Detection Reagent was added to each well and incubated at 25 °C for 40 min. Finally, kinase buffer (40 mM Tris, pH 7.5; 20 mM MgCl_2_; 0.1 mg/mL BSA; 50 μM DTT) was added and luminescence was recorded (Integration time 0.5 s). This experiment was repeated at least three times. 

### 3.8. Molecular Dynamic Simulation

The structural stability and conformational flexibility of the docked complex structures was evaluated by molecular dynamics (MD) simulations using GROMACS MD package (v2019.1) with Charmm36-jul2021 force field [34]. The CHARMM General Force Field (CGenFF, https://cgenff.umaryland.edu/) program was applied to parameterize the required topologies, atomic types, and charges for the candidate compounds. The output files of the ligand and the generated GROMACS compatible files for proteins were merged by using an ad hoc script. The protein–ligand complexes were taken as an initial structure for MD simulation. The whole system was placed at the center of a cubic box filled with water molecules (TIP3P water model) followed by further charge stabilization using Cl^−^ and Na^+^ (0.154 M concentration). Energy minimization was carried out for 100 ps using a maximum force ≥10.0 kJ mol^−1^ to obtain stable system. Subsequently, the systems were heated up from 0 to 300 K with the restraint on the solute in the NVT ensemble, then equilibrated in NPT ensemble with a temperature of 300 K and a pressure of 1 bar. The entire trajectories were saved for analysis at a frequency of 100 ps during the 100 ns MD simulation running. Various parameters were applied to analyze the results of molecular dynamics simulation, such as RMSD, root-mean-square fluctuation (RMSF), radius of gyration (Rg), trajectory clustering, principal component analysis (PCA), secondary structure (SS), solvent accessible surface area (SASA), tertiary contact map, hydrogen bond number (H-bond), and binding modes. The trajectories were analyzed using GROMACS inbuilt tools, PyMOL v2.5.0, and g_mmpbsa programs. 

### 3.9. Binding Free-Energy Calculation 

Molecular Mechanics/Poisson-Boltzmann Surface Area (MM-PBSA) is an effective method in CADD to measure binding free energy (ΔG_bind_) [35]. A total of 50 snapshots were extracted from the last 5 ns MD at 100 ps intervals for binding free-energy (ΔG_bind_) calculation using g_mmpbsa method. The binding free energy was comprised of van der Waals (ΔG_vdw_), electrostatic (ΔG_ele_), polar (ΔG_pol_), and nonpolar solvent (ΔG_nonpol_) solvation energy. 

## 4. Conclusions

In this investigation, virtual screening methods, including pharmacophore models, Bayesian classifiers, and molecular docking, were applied to discover novel inhibitors of SGK1 from the database with 29,158 compounds. Then, the screened compounds were subjected to ADME/T, PAINS, and drug-likeness analysis. Finally, 28 compounds with potential inhibition activity against SGK1 were selected for biological evaluation. The kinase inhibition activity test revealed that among these 28 hits, hit15 exhibited the highest inhibition activity against SGK1, which gave a 44.79% inhibition rate at the concentration of 10 µM. In order to further investigate the interaction mechanism of hit15 and SGK1 at simulated physiological conditions, a molecular dynamics simulation was performed. The molecular dynamics simulation demonstrated that hit15 could bind to the active site of SGK1 and form stable interactions with some key residues, such as Tyr178, Ile179, and Val112. The binding free energy of SGK1-hit15 was −48.90 kJ·mol^−1^. Therefore, the identified hit15 with novel scaffold may be a promising lead compound for the development of new SGK1 inhibitor for various diseases treatment.

## Figures and Tables

**Table 1:** The related parameters of the Hypo1 and Hypo2 for the decoy set.

Parameter	Hypo 1 Results	Hypo 2 Results
Total molecules in database (*D*)	1000	1000
Total number of actives in database (*A*)	119	119
Total hits returned (*Ht*)	135	118
Active hits returned (*Ha*)	97	92
% Yield of actives [(*Ha/Ht*) × 100]	71.85%	77.97%
% Ratio of actives [(*Ha/A*) × 100]	81.51%	77.31%
Enrichment factor (E) [(*Ha* × *D*)/(*Ht* × *A*)]	6.04	6.55
False negatives [*A*–*Ha*]	22	27
False positives [*Ht*–*Ha*]	38	26
Goodness of hit score (*GH*) *	0.71	0.76

* Güner-Henry.

**Table 2:** RMSD values of the native ligands between the crystallized and docked conformations.

PDB ID	RMSD Values ^a^				
	Ligand	AutoDock	AutoDock Vina	GOLD	CODCKER
3HDM	GMG	0.67	3.32	0.70	0.43
3HDN	MMG	0.56	3.53	1.68	4.23
Average RMSD		0.62	3.42	1.19	2.33

Abbreviation: RMSD, root-mean-square deviation. ^a^ The root of the mean square distance between the docked poses and their corresponding bound conformations (lower RMSD values indicate the program has better docking effect for this study).

**Table 3:** Performance of the Bayesian models based on the docking scores of each single representative complex and ensembles in the training set and test set.

Model Name		Roc Score	TP	FN	SE	FP	TN	SP	*Q*	MCC
3HDM_96970	Training	0.88	82	13	0.86	158	647	0.80	0.81	0.46
	Test	0.91	22	2	0.92	29	147	0.84	0.85	0.56
3HDN_90390	Training	0.90	83	12	0.87	155	650	0.81	0.81	0.48
	Test	0.94	23	1	0.96	33	143	0.81	0.83	0.56
3HDN_97440	Training	0.88	80	15	0.84	147	658	0.82	0.82	0.47
	Test	0.92	22	2	0.92	29	147	0.84	0.85	0.56
3HDN_75960	Training	0.90	83	12	0.87	157	649	0.81	0.81	0.47
	Test	0.93	24	0	1.00	34	142	0.81	0.83	0.58
Model_ensemble	Training	0.93	84	11	0.88	123	582	0.83	0.83	0.52
	Test	0.95	24	0	1.00	28	148	0.84	0.86	0.62

## Data Availability

Not applicable.

## Supplementary Materials

The following supporting information can be downloaded at: www.mdpi.com/article/10.3390/ijms23158635/s1.
